# *Stomatalia*: a deep learning-based platform for quantitative stomata and pavement cell analysis

**DOI:** 10.1186/s13007-026-01568-9

**Published:** 2026-07-07

**Authors:** Dan Jeric Arcega Rustia, Marcello Gazale, Micha Gracianna Devi, Gerard van der Linden, Lucia Sandra Perez-Borroto

**Affiliations:** 1 Greenhouse Horticulture, Wageningen Plant Research, Wageningen University & Research, Droevendalsesteeg 1, 6708PB Wageningen, The Netherlands; 2 Plant Breeding, Wageningen University & Research, Droevendalsesteeg 1, 6708PB Wageningen, The Netherlands

**Keywords:** Deep learning, Digital phenotyping, Pavement cells, Stomata

## Abstract

**Background:**

Stomata and pavement cells are fundamental components of the leaf epidermis, jointly regulating gas exchange, water loss, and leaf surface expansion. Stomata size, aperture, density, and pavement cell morphology are critical parameters for assessing plant transpiration efficiency, epidermal growth dynamics, and adaptive responses to environmental constraints. Despite their biological importance, quantifying stomatal and pavement-cell traits remains seldom not generalized, simple, and fast enough . Manual or semi-automated approaches limit large-scale phenotyping and restrict the integration of epidermal morphology into crop-improvement pipelines aimed at developing climate-resilient varieties with optimised stomatal patterning. To address such limitations, we developed *Stomatalia*, a deep learning-based platform designed to automate and standardise the quantification of stomatal and pavement cell traits. The algorithm was trained on epidermal images of cultivated and wild potato and tomato genotypes grown under optimal and abiotic-stress conditions. *Stomatalia* automatically detects stomata and pavement cells and extracts a broad range of morphological and integrative epidermal parameters, enabling high-throughput phenotyping within a unified workflow.

**Results:**

Prior to platform development, we optimised a rapid, minimally-destructive leaf-printing protocol that generates negative impressions of the leaf surface within 40–100 s. Transparent positive prints were subsequently produced and imaged under a light microscope at scale settings ranging from 20 to 200 μm. The resulting images are analysed using *Stomatalia’s* user-friendly web-based interface, which runs an instance-segmentation deep learning algorithm to detect, count, and calculate stomatal and pavement cell parameters. The platform outputs structured files containing raw measurements, derived integrative traits, and associated metadata, facilitating downstream statistical and physiological analyses. Algorithm evaluation on independent datasets demonstrated high performance within the validated dicot imaging domain, with *F*_*1*_-scores ranging from 0.86 to 0.94 depending on image scale, species, and resolution, and high segmentation overlap for both stomata and pavement cells. The generality of stomatal detection was also tested on spring onion, chickpea, balsam poplar, and wheat in cross-species feasibility tests, although performance was more variable in monocots, and pavement-cell segmentation remained species- and architecture-dependent. Benchmarking against another publicly available app further showed that, under the tested web interface settings and image types, *Stomatalia* exhibited closer agreement with manual counts and substantially faster processing times. The practical performance of *Stomatalia* was further tested in a proof-of-concept trial with potato plants subjected to optimal irrigation and a long, gradual drought. The platform reliably quantified epidermal traits despite variations in leaf morphology and image quality, supporting the integrated interpretation of stomatal and pavement-cell responses under stress.

**Conclusions:**

We developed *Stomatalia* as a robust, user-friendly deep learning platform for automated, high-throughput analysis of bright-field leaf epidermal images across varying magnifications and resolutions. *Stomatalia* facilitates rapid, reproducible, and coordinated phenotyping of stomatal and pavement cells by integrating methodological standardisation, computational automation, and multi-trait extraction in a single analytical workflow. Its strongest current application is the analysis of high-quality dicot leaf-print images, particularly in species and imaging conditions similar to those used for model training and validation. Cross-species and benchmark analyses further define its current scope: stomatal detection can be transferred to some additional epidermal architectures, whereas robust pavement-cell segmentation in monocots or highly divergent species will require further annotation and model retraining. Within these defined boundaries, *Stomatalia* provides a flexible and extensible framework for studying stomatal and pavement cell morphology and environmental plasticity, while also supporting broader efforts to dissect and optimise plant responses to abiotic stress.

**Supplementary Information:**

The online version contains supplementary material available at https://doi.org/10.1186/s13007-026-01568-9.

## Background

High-throughput phenotyping (HTP) tools are urgently needed to accelerate the development of climate-resilient crops in response to increasingly challenging environments. Most technological advancements in this field focus on traits measured at the crop or whole-plant level. Consequently, the number of available tools for analysing plant organ-level traits with comparable precision remains limited.

At the leaf level, the coordinated behaviour of stomata and pavement cells plays a central role in regulating plant productivity and stress adaptation. Stomata control CO₂ uptake for photosynthesis while simultaneously regulating water loss through transpiration, thereby determining intrinsic water-use efficiency [12]. Pavement cells, in turn, govern epidermal expansion and mechanical properties of the leaf surface, directly influencing leaf growth dynamics and surface area development [15]. Understanding the structural and functional interplay between these two epidermal cell types is therefore essential to elucidating plant evolutionary advantages in gas-exchange efficiency, water-use optimisation, leaf development, and adaptive plasticity under environmental stresses.

Importantly, stomatal size, aperture, density, and pavement cell morphology are critical parameters for investigating plant responses and adaptations to environmental stresses. However, quantifying these traits remains time-intensive and low-throughput, particularly when analysing large datasets or plants exposed to environmental conditions that alter leaf morphology (e.g., reduced leaf size, wrinkling, rolling, or wilting).

A key biological limitation of many current studies is that stomatal traits are frequently interpreted in isolation, typically normalised to leaf area alone. Because stomatal density is intrinsically linked to epidermal expansion, failing to integrate pavement cell morphology can distort interpretation. For example, an apparent increase in stomatal density under drought may simply reflect reduced leaf expansion rather than an active developmental adjustment. Thus, the absence of pavement cell morphology analysis risks introducing bias into stress-response studies.

Several computational tools have been developed to facilitate the quantification of leaf morphological traits. Advances in image processing and deep learning have enabled workflows for analysing leaf surface characteristics [9], leading to the development of platforms such as Cellpose, PlantSeg, PaCeQuant, StomataCounter, LeafNet, MorphoGraphX, and SCAN [1, 5, 10, 13, 18, 21, 23]. These tools efficiently quantify object number and size, and although more integrative approaches have been proposed, including algorithms for detection, counting, and index-based trait integration [24], they generally do not provide integrative parameters coupling stomatal and pavement cell traits, such as density relative to epidermal expansion or composite surface indices [6]. In addition, available tools differ in their intended image types, acquisition requirements, and biological scope, making it important to evaluate new workflows not only within their primary training domains but also across species, epidermal architectures, and existing analysis platforms.

From a methodological perspective, most available algorithms rely on object detection and/or semantic segmentation. Object detection typically approximates targets with rectangular bounding boxes, which are ill-suited to irregularly shaped structures such as stomata and pavement cells, thereby limiting morphological precision. Semantic segmentation, while pixel-accurate, does not distinguish between individual objects of the same class, preventing *per*-cell trait extraction. A more suitable approach is to use instance segmentation, a single model that combines detection and segmentation, enabling accurate delineation and quantification of individual cells.

To address the above-mentioned biological and methodological gaps, we developed and validated *Stomatalia*, a deep learning-based platform capable of annotating, quantifying, and integrating stomatal and pavement cell morphological traits. The algorithm was trained on cultivated and wild tomato and potato accessions sampled under optimal and environmentally stressful conditions and is designed to remain robust when analysing images of stressed plants exhibiting altered leaf morphology. Because model performance can depend on image quality, species identity, and epidermal architecture, we further evaluated *Stomatalia* across imaging scales, in-house and external cross-species image sets, and benchmarked its practical performance against an existing integrated epidermal-analysis workflow.

This study aims to present a new, comprehensive workflow for the automated analysis of stomatal and pavement cells. The objectives of the study were to (1) develop a fast, accurate, and integrative instance segmentation algorithm for stomata and pavement cell trait extraction; (2) design an intuitive web-based platform to facilitate accessibility and reproducibility; (3) evaluate robustness across imaging settings, species, and epidermal architectures; (4) benchmark the advantages and limitations of this workflow relative to existing approaches; and (5) provide proof of concept by investigating physiological responses to drought through integrated stomatal and pavement cell analysis. Together, these objectives aim to improve analytical precision, throughput, and biological interpretability, thereby advancing stomata and pavement cell phenotyping as a key component of research on plant physiology and crop resilience.

## Methods

### Plant material and growth conditions for model training

Images used for model training were obtained from the tomato species *Solanum habrochaites* (LA1777) and *S. lycopersicum* cv. Moneymaker, the wild potato *Solanum berthaultii* (BER481-3) and the cultivated *Solanum tuberosum* (L.) cv. RH89-039-16. Tomato seeds were sown directly in a soil–peat substrate (Lensli^®^), while potato genotypes were propagated in vitro and, after two weeks, transferred to 17-cm pots containing potting soil. All plants were grown in a climate-controlled greenhouse under a 16 h light/8 h dark photoperiod, with an average PAR of 500 µmol m^− 2^ s^− 1^, day/night temperatures of 22 °C/19°C, and 70% relative humidity. Irrigation was maintained at approximately 60% of field capacity throughout the experiments.

### Leaf print preparation and image acquisition

**Fig. 1:**
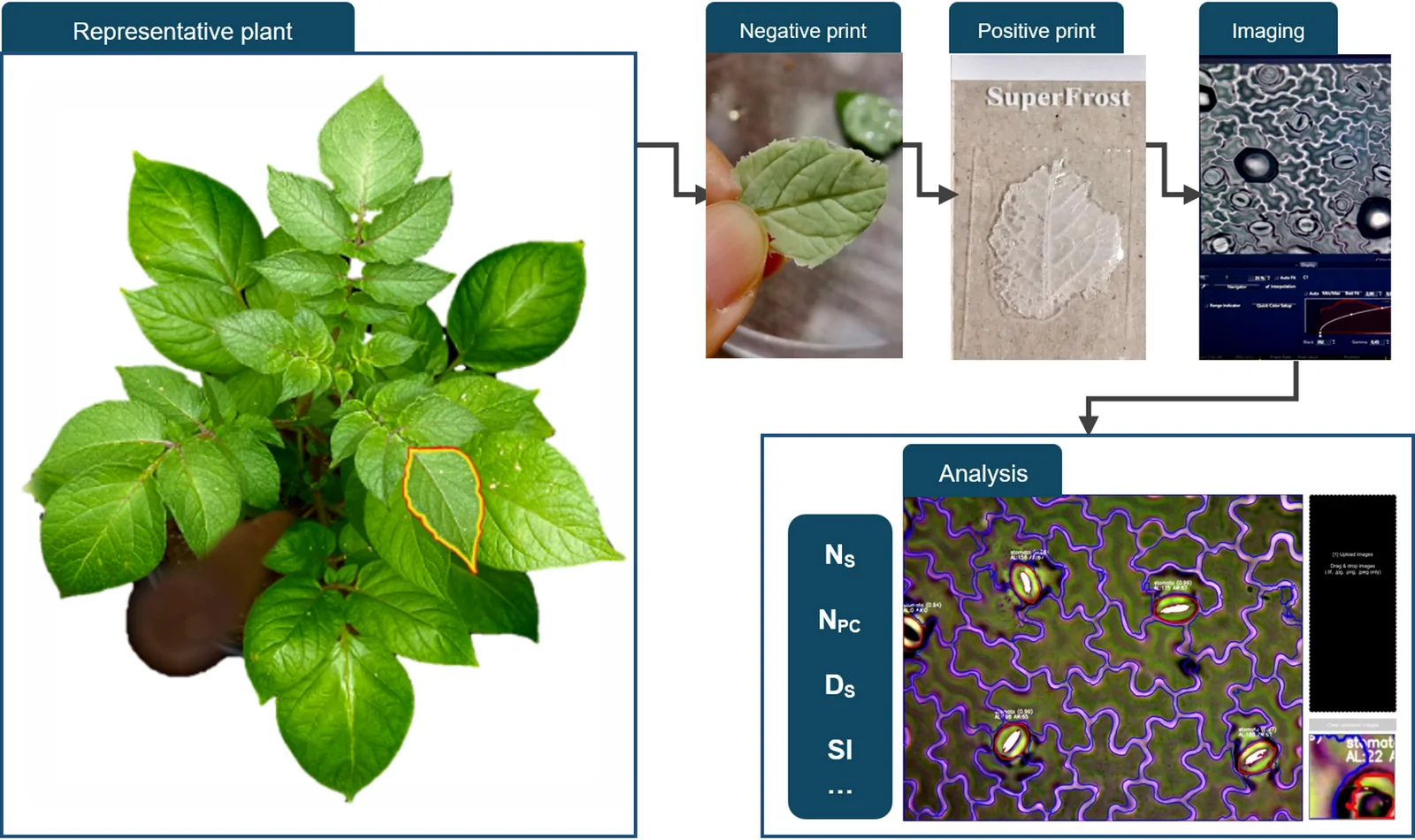
Workflow illustrating plant sampling, generation of negative and positive leaf impressions, microscopic imaging, and subsequent analysis using the *Stomatalia* platform. Output parameters include stomatal number (N_S_), pavement cell number (N_PC_), stomatal density (D_S_), stomatal index (SI), and additional integrative epidermal traits

The acquisition method described in this section was used to generate all the images used to train, validate and test *Stomatalia*. Leaf epidermal prints were obtained from both adaxial and abaxial leaf surfaces of 3-4-week-old plants. For potato and tomato, impressions were acquired from the central leaflet of the third youngest fully unfolded leaf (Fig. 1).

Negative leaf impressions were generated by applying a uniform layer of dental resin (PRESIDENT The Original Light Body, Coltene), prepared at a 2:1 base-to-catalyst ratio, directly onto the leaf surface. This fast-curing resin was preferred over alternative approaches, such as direct nail polish impressions or epidermal peels. Direct application of nail polish requires longer drying times and involves volatile solvents that may induce rapid changes in stomatal aperture during sampling. Epidermal peels, while useful for some applications, are more labour-intensive, destructive, and less suitable for high-throughput phenotyping. In contrast, the use of dental resin facilitated rapid impression acquisition while reducing the time of chemical exposure to the leaf surface.

To minimise mechanical disturbance and reduce the risk of trichome removal, the resin was gently spread in a single direction and cured for approximately 1.5 min. Then, resin moulds were carefully peeled from the leaf surface and stored in labelled paper envelopes. Although this method is considered minimally destructive, minor mechanical disturbances cannot be entirely excluded, including trichome removal, local surface damage, or residual resin traces. These factors could potentially affect epidermal integrity or stomatal behaviour. Therefore, repeated sampling of the same leaf surface should be avoided.

Positive leaf prints were subsequently produced by coating the cured silicone moulds with a thick layer of transparent nail polish (60 s SuperShine, Rimmel London). Once fully dried, the positive impressions were mounted on clear microscope slides (SuperFrost^®^, Menzel Gläser), secured with transparent adhesive tape, labelled, and imaged using a ZEISS light microscope equipped with an AxioCam IC camera. Images were acquired at 37.8× magnification, corresponding to a 0.63× camera adapter and 60× objective. For each leaf print, four non-overlapping fields of view were captured. Depending on the dataset, images were acquired at scales corresponding to 20 μm, 50 μm, or 100 μm scale bars, including additional quality-test images at 200 μm.

### Algorithm for automatic stomata and pavement cell analysis

**Fig. 2:**
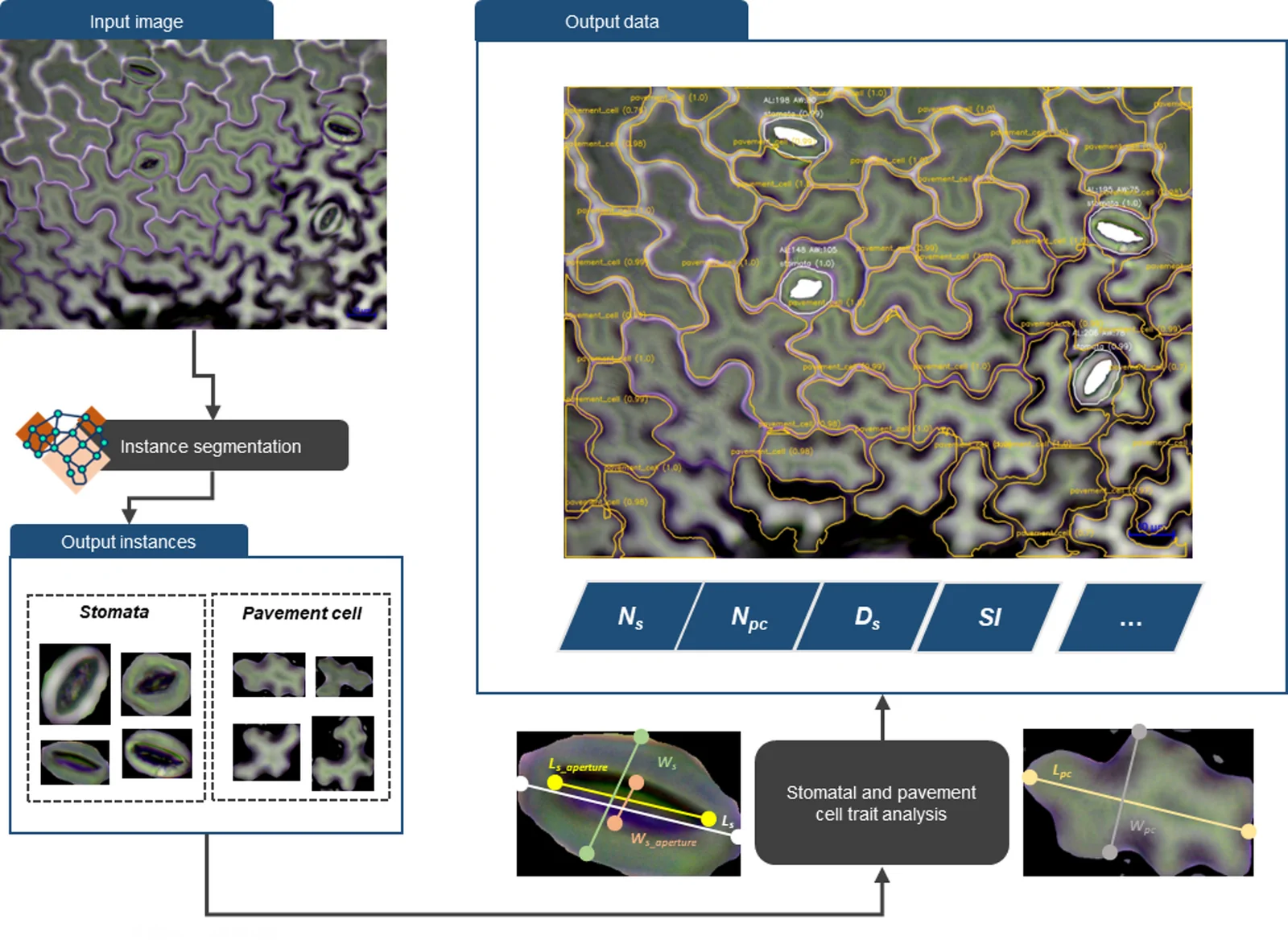
Schematic representation of the *Stomatalia* analysis pipeline. Input epidermal images are processed by an instance segmentation model to detect and delineate individual stomata and pavement cells. Segmented instances are subsequently quantified to extract morphological traits, including stomatal number (N_S_), pavement cell number (N_PC_), stomatal density (D_S_), stomatal index (SI), aperture-related parameters, and additional integrative epidermal metrics

The algorithm for automated stomata and pavement cell analysis (Fig. 2) was developed in Python (v3.8.3) using the OpenCV library (v4.10.0) for image processing, PyTorch (v1.12) for deep learning implementation, and the Detectron2 framework (v0.6) for instance segmentation. The model simultaneously detects and segments individual stomata and pavement cells, enabling *per*-cell extraction of morphological traits.

The overall workflow comprises (i) dataset preparation and annotation, (ii) instance segmentation model training and optimisation, and (iii) automated extraction of stomatal and pavement cell morphological traits from segmented objects. Details of dataset preparation and algorithmic procedures are described in further sections. All computations were conducted on a workstation running Ubuntu 22.04, equipped with an Intel Xeon E5-1650 CPU, an NVIDIA GeForce GTX Titan X GPU, and 16 GB RAM.

#### Dataset preparation

Leaf print images were annotated using Darwin v7 (V7 Labs). Two object classes were defined: stomata and pavement cells. Individual objects were manually annotated by drawing polygonal masks along their boundaries to enable instance segmentation training.

The training and validation datasets were generated independently. The training dataset consisted of tomato and potato leaf-print images acquired at two magnification scales, corresponding to 20 μm and 50 μm scale bars. Images acquired at the 20 μm scale provided higher magnification and finer structural detail, whereas images acquired at the 50 μm scale captured larger epidermal areas containing more stomata and pavement cells, but with reduced per-cell detail. The validation datasets were generated from subsequent experiments using wild and cultivated tomato and potato accessions. These validation images were acquired from independent leaf prints and microscopy sessions and were not used during model training. Validation images were collected at 20 μm and 50 μm scales to evaluate model performance across different imaging settings and biological materials. No leaf print, microscopy acquisition series or image was shared between the training and validation datasets.

A statistical summary of the training and validation datasets is provided in Table 1. A subset of validation images, corresponding detection outputs, and exported datasets are available at https://doi.org/10.6084/m9.figshare.32532672, to facilitate independent inspection of *Stomatalia* outputs.

**Table 1:** Training and validation dataset summary

Purpose	Scale	No. of images	*N*_S_	*N*_PC_	Crop(s)
Training	20 μm	132	1469	9675	Tomato, potato
Training	50 μm	132	2346	18,399	Tomato, potato
Validation	20 μm	287	5536	22,518	Tomato
Validation	50 μm	227	4722	27,483	Potato

Scale refers to the microscope/image acquisition scale used for the corresponding dataset. Images were exported at 2560 × 1920 px

N_S_: number of stomata; N_PC_: number of pavement cells

#### Instance segmentation

Stomatal and pavement cell detection was formulated as an instance segmentation task, in which each object is identified by both its bounding box coordinates and a pixel-wise segmentation mask. Instance segmentation enables precise delineation of individual objects, facilitating accurate extraction of *per*-cell morphological traits.

Several models were compared in this work: Mask Region-Based Convolutional Neural Network (Mask R-CNN) [7], Mask R-CNN with Point-based Rendering (PointRend) [8], YOLOv8 [16], and YOLOv9 [20]. Mask R-CNN extends Faster R-CNN by incorporating an additional branch that predicts a binary segmentation mask for each detected region of interest. Each pixel in the binary mask indicates whether a detected object (foreground) is present or not (background). Meanwhile, Mask R-CNN + PointRend further improves segmentation by iteratively refining coarse mask predictions via point-wise feature sampling, enabling higher boundary precision. This refinement is particularly relevant for stomata and pavement cells, whose irregular contours require fine-grained segmentation for accurate morphological measurements. Finally, YOLOv8 and YOLOv9 are recent real-time instance segmentation architectures optimised for speed and efficiency.

For training and evaluation, Mask R-CNN-based models were configured with an input size of 1600 × 1600 pixels, whereas YOLO-based models used 960 × 960 pixels. All models were initialised with weights pretrained on the COCO dataset and implemented using their respective deep learning frameworks.

#### Stomatal and pavement cell trait acquisition

Following instance segmentation, detected objects were separated into stomatal and pavement cell classes, as shown in Fig. 2. Bounding box coordinates of each detected object were used to crop individual stomata or pavement cells (objects), and segmentation masks were applied to isolate foreground pixels from the background. Morphological traits were computed from object contours. The rotating callipers algorithm [17] was applied to each contour to estimate the major and minor axes. For stomata, the major axis was defined as stomatal pore length *S*_*PL*_, and the minor axis as stomatal pore width *S*_*PW*_. For pavement cells, analogous measurements were defined as pavement cell length *PC*_*L*_ and pavement cell width *PC*_*W*_.

**Fig. 3:**
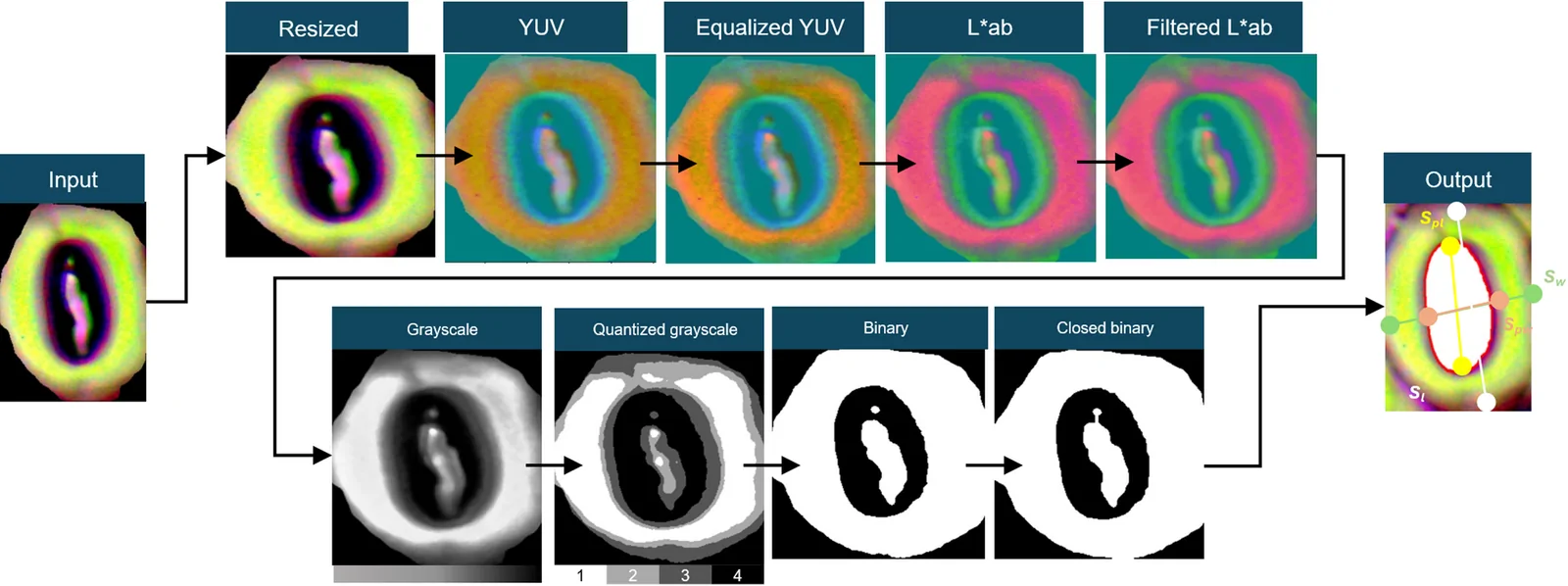
Workflow of the stomatal pore extraction algorithm. After instance segmentation and object cropping, colour-space transformation, contrast enhancement, filtering, quantisation, and morphological operations are applied to isolate the stomatal pore contour for pore-aperture assessment

To extract the stomatal pore region, each detected stomatal instance was first resized to 256 × 256 pixels (Fig. 3).

The cropped RGB image was converted to the YUV colour space, and histogram equalisation was applied to the V channel to enhance contrast. The image was then converted to the L*a*b* colour space, followed by median filtering (kernel size = 5) to reduce noise. The filtered image was converted to grayscale and quantised by regrouping pixel intensities (0–255) into four discrete levels. Binary thresholding was subsequently applied, grouping levels 1–3 as foreground and level 4 as background. Morphological closing was performed to ensure continuity of the pore region. Finally, contour detection was applied to the processed binary image, while retaining only the child contours, such as the stomata pore depicted by a red line in Fig. 3.

#### Dimensional conversion

Pixel-based measurements were converted into real-world units (µm or µm²) using a dimensional calibration procedure. A reference scale was drawn on a representative image using the microscope software to obtain a known distance in µm. The number of pixels corresponding to this reference distance was manually determined. The pixel-to-micrometre conversion ratio was calculated using Eq. 1.

1 $$\:Pixel\:ratio=\:\frac{known\:distance\:\left(\mu\:m\right)}{reference\:\#\:of\:pixels}$$

#### Quantified traits

The algorithm extracts stomatal and pavement cell traits summarised in Table 2.

**Table 2:** List of traits measured by the algorithm

Symbol	Parameter	Unit	Description/ Formula
S_n_	Stomata number	-	S_n_ = ∑S; S= Detected stomata
PC_n_	Pavement cell number	-	PC_n_ = ∑PC; PC= Detected pavement cells
SP_l_	Stomata pore length	µm	Length of the area between guard cells
SP_w_	Stomata pore width	µm	Width of the area between guard cells
SA_a_	Mean normalized stomata aperture	µm	SA_a_= ∑SA/S_n_; SA = SP_w_/SP_l_
SA_sd_	SD of normalized stomata aperture	µm	SA_sd_ = √(∑(SA − SA_a_)^2^ /S_n_); proxy to detect different rates of stomata closure
SS_a_	Mean stomata size	µm^2^	SS_a_= ∑SS/S_n_; SS= area of stomata
SS_sd_	SD of stomata size	µm^2^	SS_sd_ = √(∑(SS − SS_a_)^2^ /S_n_); proxy to detect different lineages of stomata
SD	Stomatal density	µm^2^	SD = ∑S_n_/OA; OA= Observation area (µm^2^); density per total image area
SI	Stomatal index	%	SI = (S_n_/ S_n_+PC_n_)*100; density per total PC area
TSA	Transpiration surface area	µm^2^	TSA = ∑SA; area of the leaf covered by stomata
PCS_a_	Mean pavement cell size	µm^2^	PCS_a_= ∑PCS/PC_n_; PCS= area of pavement cell
PCS_sd_	SD of pavement cell size	µm^2^	PCS_sd_ = √(∑(PCS−PCS_a_)^2^ /PC_n_); proxy to detect leaf expansion

*Unit depends on reporting conversion

### Algorithm testing

#### Detection and segmentation

The instance segmentation models were evaluated using detection and segmentation metrics. In both sets of metrics, Intersection over Union (*IoU*) was used for evaluation. To distinguish between the evaluation of two metrics, *IoU* for object detection is denoted *IoU*_*det*,_ and *IoU* for segmentation is denoted *IoU*_*seg*_. *IoU* ranges from 0 to 1.0, with values closer to 1.0 indicating greater overlap. *IoU*_*det*_ is computed as shown in Eq. 2.

2 $$\:{IoU}_{det}=\:\frac{area({b}_{pred}\cap\:{b}_{gt})}{area({b}_{pred}\cup\:{b}_{gt})}$$

where *b*_*pred*_ and *b*_*gt*_ represent predicted and ground-truth bounding boxes, respectively.

If the *IoU*_*det*_ of two compared objects was above 0.5, then it was considered as a matched detection. True positives (*TP*_*det*_), false positives (*FP*_*det*_) and false negatives (*FN*_*det*_) were detected and used to compute precision, recall, and *F*_1_-score, as shown in Eq. 3 to 5.

3 $$\:Precision=\:\frac{{TP}_{det}}{{TP}_{det}+{FP}_{det}}$$

4 $$\:Recall=\:\frac{{TP}_{det}}{{TP}_{det}+{FN}_{det}}$$

5 $$\:{F}_{1}{\text{ - }}score=\:\frac{2 * precision * recall}{precision+recall}$$

Segmentation accuracy was evaluated using pixel-level Intersection over Union (*IoU*_*seg*_):

6 $$\:{IoU}_{seg}=\:\frac{{TP}_{seg}}{{TP}_{seg}+{{FP}_{seg}+FN}_{seg}}$$

where *TP*_*seg*_, *FP*_*seg*_, and *FN*_*seg*_ represent true positive, false positive, and false negative pixels, respectively, after matching predicted and ground-truth masks. *IoU* values range from 0 to 1, with values closer to 1 indicating better overlap between predictions and ground truth.

#### Model generalisation across various imaging settings and species

To evaluate the robustness and generalisation capacity of *Stomatalia*, we assembled independent test datasets covering differences in image scale, image quality, species identity, and epidermal architecture. A statistical summary of the test datasets is presented in Table 3.

First, a scale test was performed using independent potato and tomato leaf-print images that were generated at 20, 50, and 100 μm magnification settings. For each scale, 15 images were acquired and analysed. The images were used to evaluate whether the algorithm could maintain stomatal and pavement-cell detection across changes in field of view, cell size representation, and object density.

Second, to assess sensitivity to image quality, the algorithm was further tested on a subset of five images of varying quality, including purposely sharp and blurred images at scales of 20, 50, 100, and 200 μm.

Lastly, a cross-species test was conducted using monocot and dicot epidermal images from to assess *Stomatalia’s* transferability beyond the main dicot training dataset. A set of 15 leaf-print images from five-week-old spring onion plants (*Allium fistulosum* cv. Performer), acquired at 20 μm, was generated in-house and tested. Additional external images were obtained from the StomataCounter repository hosted on Roboflow (https://universe.roboflow.com) and included nine images of chickpea (*Cicer arietinum*), nine leaf-print images of *Arabidopsis thaliana* ecotype Columbia-0, nine images of maize (*Zea mays*), six images of balsam poplar (*Populus balsamifera*), and eight leaf-print images of common wheat (*Triticum aestivum*). Together, these datasets allowed us to examine the behaviour of *Stomatalia* across epidermal types with different pavement-cell morphology, stomatal *organisation*, and monocot versus dicot leaf architectures.

All test images were analysed using the same *Stomatalia* workflow. Images acquired in-house were exported at 2560 × 1920 pixels, whereas external datasets were analysed at the source repository image resolution, ranging from 2048 × 1536 to 3840 × 2748 pixels. The potato, tomato, and spring onion datasets served as independent in-house test datasets for quantitative evaluation of the algorithm. For these images, manual instance-level annotations were available, allowing calculation of detection metrics, including *F*_*1*_-score and miss rate, as well as segmentation accuracy using *IoU* for stomata and pavement cells. In contrast, the external cross-species datasets were used as feasibility and domain-transfer tests. For these images, manual ground-truth counts were generated in Fiji (ImageJ2 v. 2.14), enabling calculation of count-based *F*_*1*_-scores and miss rates, but not pixel-level segmentation *IoU*, as instance-level mask overlap was unavailable.

**Table 3:** Summary of independent test datasets used to evaluate *Stomatalia* across imaging scales, image quality, and species

Scale	Resolution, px	No. of images	*N*_S_	*N*_PC_	Classification	Crop/species	Type of test(s)	Source
20 μm	2560 × 1920	15	176	1171	Dicot	Tomato	Scale, image quality	In-house
20 μm	2560 × 1920	15	85	1	Monocot	Spring onion	Cross-species	In-house
50 μm	2560 × 1920	15	562	2762	Dicot	Potato	Scale, image quality	In-house
100 μm	2560 × 1920	15	608	3007	Dicot	Tomato	Scale, image quality	In-house
20 μm	2560 × 1920	9	157	287	Dicot	Chickpea	Cross-species	StomataCounter
20 μm	2048 × 1536	9	136	672	Dicot	*A. thaliana* Col-0	Cross-species	StomataCounter
20 μm	2048 × 2048	6	208	51	Dicot	Balsam poplar	Cross-species	StomataCounter
50 μm	3840 × 2748	9	18	1095	Monocot	Maize	Cross-species	StomataCounter
50 μm	2592 × 1944	8	83	1	Monocot	Wheat	Cross-species	StomataCounter

Scale refers to the microscope/image acquisition scale used for the corresponding dataset. Images acquired in-house were exported at 2560 × 1920 px, whereas external images were analysed at the resolution provided by the source repository

N_S_: number of stomata; N_PC_: number of pavement cells

#### *Stomatalia *benchmarking against other open-source tools

To assess the comparability of *Stomatalia* with other epidermal analysis tools, an external benchmark was conducted using 27 independent test images, analysed with both the *Stomatalia* and LeafNet web interfaces (Table 4), different from the dataset shown in Table 3. LeafNet was selected because it is the available platform that most closely overlaps with *Stomatalia*, as it supports stomata and pavement-cell detection and segmentation in bright-field leaf epidermal images. In the original LeafNet study, the platform was benchmarked against several established tools, including StomataCounter, PaCeQuant, MorphoGraphX, PlantSeg, and Cellpose, and showed strong performance across the stomata and pavement-cell detection and segmentation tasks [10]. Therefore, LeafNet was considered the most suitable comparator for evaluating *Stomatalia* against an existing integrated epidermal-analysis workflow.

**Table 4:** Summary of independent test dataset used to benchmark *Stomatalia* against LeafNet

Scale	Dimensions	No. of images	*N*_S_	*N*_PC_	Crop/species	LeafNet parameter
20 μm	2560 × 1920	9	106	637	Potato	Default; 2.17 px/µm resolution
20 μm	640 × 480	9	49	-	Spring onion	Default; 0.54 px/µm resolution
20 μm	2048 × 1536	9	136	672	*A. thaliana* Col-0	Default; 1.74 px/µm resolution
20 μm	2560 × 1920	3	41	231	Potato	Default; 2.17 px/µm resolution; no denoising
20 μm	640 × 480	3	15	-	Spring onion	Default; 0.54 px/µm resolution; no denoising

Values correspond to *Stomatalia *detections in each image set. The dash indicates that pavement-cell detection was not considered for spring onion images, as this species exhibits a monocot epidermal architecture outside the main pavement-cell training domain of *Stomatalia*

N_S_: number of stomata; N_PC_: number of pavement cells

The test image set consisted of nine potato, nine spring onion, and nine *Arabidopsis thaliana* Col-0 leaf-print images acquired at a 20 μm scale and exported at 2560 × 1920 px, 640 × 480 px, and 2048 × 1536 px, respectively. The potato set represented images within the validated dicot imaging domain of *Stomatalia*, the spring onion set represented a monocot epidermal architecture outside the main training domain, and the Col-0 images were included because LeafNet was originally trained using bright-field images of *A. thaliana* leaf surfaces. All images were first analysed in LeafNet using default parameters, including LeafSeg for pavement-cell segmentation and StomaNet Universal for stomatal detection. Image resolution was set to 2.17 px/µm for potato images, 0.54 px/µm for spring onion images, and 1.74 px/µm for Col-0 images to account for differences in export resolution. To evaluate the influence of denoising on detection outputs, a subset of three potato images and three spring onion images was reanalysed using the same image-resolution settings but with the LeafNet denoiser set to “None”. 

The LeafNet web interface does not support batch processing; therefore, images were uploaded individually and processed sequentially in the server queue. Processing time was recorded for each image to evaluate practical differences in usability between the two web-based workflows.

#### Proof-of-concept trial

To demonstrate *Stomatalia’s* applicability under biologically relevant conditions, a controlled drought experiment was conducted in a climate-controlled greenhouse at the Unifarm WUR campus. Pre-germinated seed tubers of the potato cultivars ‘Desiree’, ‘Lady Rosetta’, ‘Russet Burbank’, and ‘Spunta’ were planted in 21-cm pots filled with sand. Four weeks after planting, plants were standardised by pruning to three stems. Plants were irrigated twice daily via drip emitters, maintaining the substrate at approximately 60% of field capacity. Greenhouse climate settings were comparable to those used during image acquisition for model training. At the tuber initiation stage, drought stress was imposed by gradually reducing irrigation over a four-week period. Seventeen days after treatment initiation, the youngest fully unfolded leaf from each plant was selected for gas exchange measurements using a LI-600 porometer/fluorometer, followed by collection of epidermal imprints. Adaxial and abaxial leaf surfaces from three independent plants per genotype and treatment were sampled, and four non-overlapping images were acquired per imprint (total *n* = 192 images).

This experiment enabled evaluation of the algorithm’s scalability across a large image dataset while providing functional validation under stress conditions. Importantly, it served as a proof of concept for assessing potential interpretative bias that arises when changes in stomatal density and size are interpreted without accounting for pavement cell dynamics. In addition, comparing stomatal morphological traits with gas-exchange data enabled integration of structural and functional parameters, highlighting that assessments of plant transpiration capacity and intrinsic water use efficiency should consider stomatal dynamics alongside morphological traits.

## Results and discussion

### Model training and optimisation

To identify the most suitable architecture for deployment within *Stomatalia*, multiple instance segmentation models and backbone configurations were trained and evaluated on the independent test datasets described above. Quantitative performance metrics are summarised in Table 5.

**Table 5:** Instance segmentation model training and optimisation results

Model	Input size	*F*_1_-score	Miss rate	*IoU*_*seg*_ (stomata)	*IoU*_*seg*_ (pavement cell)	Ave. inference time (ms)
Mask R-CNN (R50)	1600 × 1600	0.94	0.09	0.88	0.87	440.2
Mask R-CNN (R101)	1600 × 1600	0.93	0.09	0.88	0.87	463.9
Mask R-CNN + PointRend (R50)	1600 × 1600	0.91	0.12	0.89	0.90	446.8
Mask R-CNN + PointRend (R101)	1600 × 1600	0.93	0.10	0.89	0.90	525.6
YOLOv8s	960 × 960	0.93	0.06	0.81	0.72	120.1
YOLOv8m	960 × 960	0.93	0.06	0.81	0.72	136.4
YOLOv9c	960 × 960	0.93	0.06	0.81	0.73	152.2

For optimisation, different Mask R-CNN backbones were evaluated, including ResNet50 (R50) and ResNet101 (R101). Additionally, two YOLOv8 configurations (small and medium) and one YOLOv9 configuration (compact) were tested.

YOLO-based models exhibited substantially faster inference times (120–152 ms) compared to Mask R-CNN-based architectures (440–526 ms). However, their segmentation performance was lower, with *IoU*_*seg*_ values ≤ 0.81 for stomata and ≤ 0.73 for pavement cells. In contrast, Mask R-CNN models achieved higher segmentation accuracy (*IoU*_*seg*_ ≈ 0.88–0.90), particularly for pavement cells, which exhibit more complex, highly lobed geometries than stomata.

Incorporating the PointRend module improved boundary refinement, resulting in slightly higher segmentation accuracy, particularly for pavement cells, despite a moderate increase in inference time. Differences between ResNet50 and ResNet101 backbones were minor in terms of detection performance, although the deeper ResNet101 backbone showed marginally improved segmentation consistency.

**Fig. 4:**
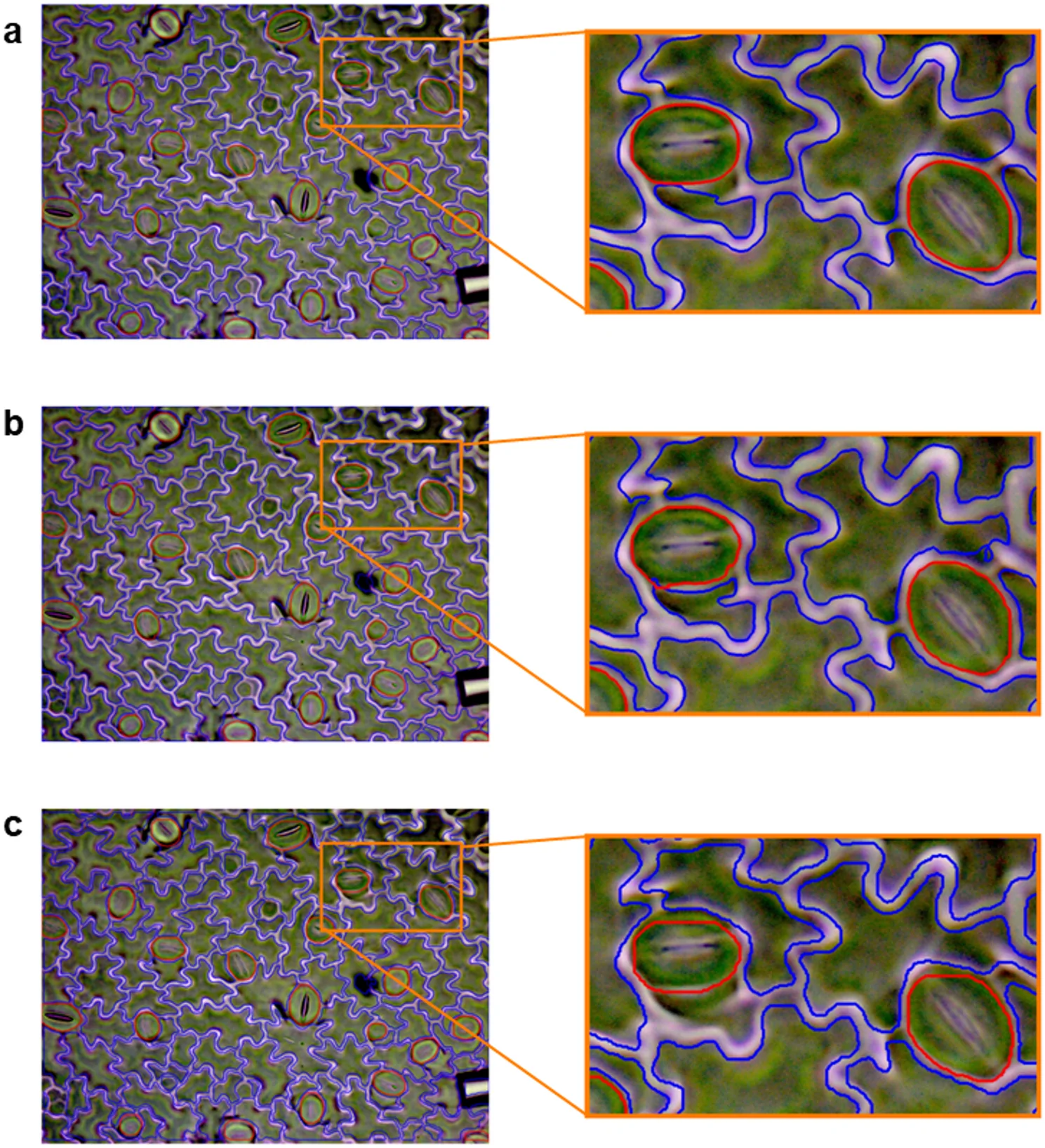
Inference results obtained using different instance segmentation models: **a** Mask R-CNN; **b** Mask R-CNN + PointRend; and **c** YOLOv9. Enlarged regions are displayed to highlight the precision of the segmentation boundary

A qualitative comparison of inference results is shown in Fig. 4.

Visual inspection corroborated the quantitative results. Mask R-CNN + PointRend more accurately captured the highly curved and interlocking contours of pavement cells, particularly in regions with pronounced cell lobing. In contrast, YOLOv9 occasionally produced truncated or clipped segmentation masks, particularly around stomatal boundaries. Furthermore, PointRend improved discrimination between adjacent pavement cells and background artefacts, enhancing contour continuity. Based on the combined quantitative metrics and qualitative assessment, the Mask R-CNN + PointRend architecture with a ResNet101 backbone was selected for deployment in *Stomatalia*, balancing segmentation accuracy and computational efficiency.

### Algorithm performance across image settings and species

**Fig. 5:**
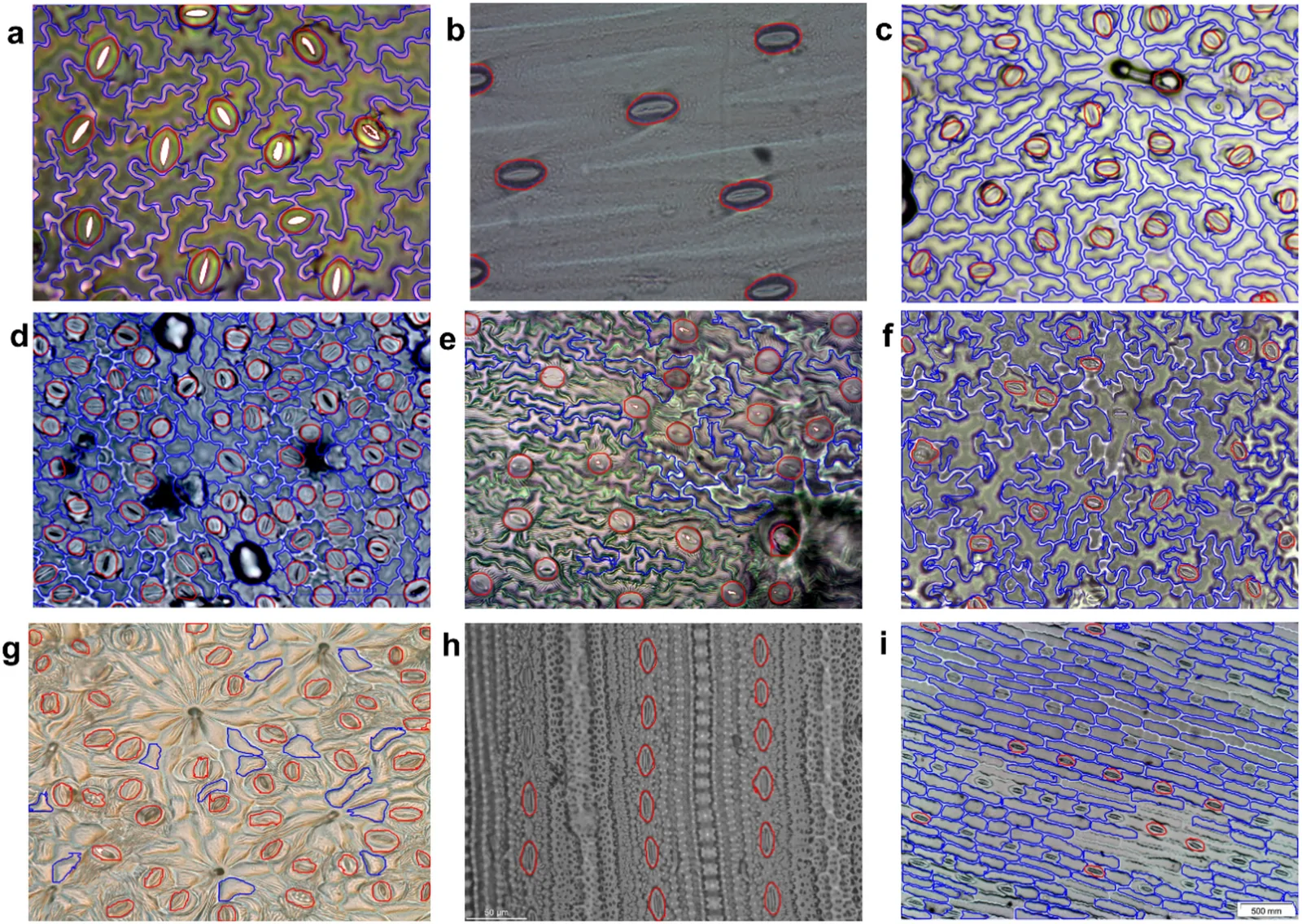
Representative *Stomatalia* output images across imaging scales and leaf types. Representative detection outputs are shown for in-house datasets: **a** tomato at 20 μm; **b** spring onion at 20 μm; **c** potato at 50 μm; **d** tomato at 100 μm; and external datasets: **e** chickpea at 20 μm; **f** *Arabidopsis thaliana* Col-0 at 20 μm; **g** balsam poplar at 20 μm; **h** wheat at 50 μm; and **i** maize at 50 μm. Red outlines indicate detected stomata, and blue outlines indicate detected pavement cells

To assess the robustness of *Stomatalia* across imaging scales and epidermal architectures, we evaluated the selected Mask R-CNN + PointRend (ResNet101) model using two complementary test sets (Table 6; Fig. 5). First, we analysed independent in-house datasets acquired at 20, 50, and 100 μm from tomato, potato, and spring onion. Second, to further explore cross-species robustness, we analysed external image sets from *Arabidopsis thaliana* Col-0, chickpea (*Cicer arietinum*), maize (*Zea mays*), balsam poplar (*Populus balsamifera*), and common wheat (*Triticum aestivum*), as defined previously in Table 3.

**Table 6:** Algorithm performance across imaging scales, quality, and cross-species

Scale	Crop/species	*F*_1_-score	Miss rate	*IoU*_*seg*_ (stomata)	IoU_seg_ (pavement cells)
20 μm	Tomato	0.94	0.06	0.94	0.92
20 μm	Spring onion	0.99	0.02	0.90	*n.a*
50 μm	Potato	0.92	0.12	0.86	0.91
100 μm	Tomato	0.86	0.22	0.85	0.87
20 μm	Chickpea	0.90	0.12	*n.a*	*n.a*
20 μm	*A. thaliana* Col-0	0.81	0.30	*n.a*	*n.a*
20 μm	Balsam poplar	0.97	0.07	*n.a*	*n.a*
50 μm	Maize	0.10	0.97	*n.a*	*n.a*
50 μm	Wheat	0.67	0.46	*n.a*	*n.a*

Instance-level metrics were calculated for in-house datasets with manual instance annotations and include F_1_-score, miss rate and segmentation IoU (IoU_seg_). External cross-species datasets were evaluated using manual ground-truth counts generated in Fiji (ImageJ2 v. 2.14); therefore, F_1_-score and miss rate for these datasets are count-based and do not represent pixel-level segmentation overlap. IoU_seg_ was therefore not calculated for the external cross-species datasets

n.a.: not available

At the 20 μm scale, the algorithm achieved high detection and segmentation performance in tomato (*F*_*1*_-score = 0.94) and spring onion (*F*_*1*_-score = 0.99), and high stomatal *IoU*_*seg*_ values in both species (Table 6; Fig. 5a, b). In tomato, pavement-cell segmentation was also accurate (*IoU*_*seg*_ = 0.92), whereas pavement-cell segmentation was not assessed in spring onion because its elongated monocot epidermal architecture falls outside the model’s main training domain (Fig. 5b). Performance gradually declined as magnification decreased, that is, as the field of view increased from 20 to 100 μm. At 50 μm in potato, performance remained high, although minor over-detection events and local pavement-cell boundary errors were observed (Fig. 5c). At 100 μm in tomato, reduced structural detail resulted in increased missed detections and a lower *F*_*1*_-score (0.86; Fig. 5d). This decline likely reflects a trade-off between spatial resolution and field coverage: larger fields of view capture more epidermal area but reduce the pixel-level representation of stomata and pavement-cell boundaries.

In the external cross-species datasets, the representative outputs further illustrated species-dependent differences in model transferability (Fig. 5e-i). Stomatal count-based performance was high in chickpea (*F*_*1*_-score = 0.90; Fig. 5e) and balsam poplar (*F*_*1*_-score = 0.97; Fig. 5g), and moderate in *A. thaliana* Col-0 (*F*_*1*_-score = 0.81; Fig. 5f) and wheat (*F*_*1*_-score = 0.67; Fig. 5h). In contrast, maize showed poor stomatal recovery, with a high miss rate of 0.97, indicating that most manually annotated stomata were not detected despite partial pavement-cell detection (Fig. 5i). Pavement-cell count-based performance was strongest in *A. thaliana* Col-0, whereas chickpea, maize, and balsam poplar showed substantial pavement-cell under-detection. Wheat pavement-cell performance could not be evaluated because image quality did not allow reliable manual pavement-cell counting.

Collectively, these results suggest that 20 μm provides the best balance between morphological resolution and field coverage for accurate trait extraction in high-quality bright-field leaf-print images. *Stomatalia* remained functional at larger fields of view, but at 50–100 μm, reduced structural detail, increased missed detections, and local segmentation errors were observed, which may influence downstream trait quantification, particularly for highly lobed pavement cells. The cross-species analyses further showed that stomatal detection can generalise beyond the tomato and potato training domain in some species, as observed for spring onion, chickpea, and balsam poplar. This likely reflects the partial conservation of stomatal morphology across diverse epidermal contexts. In contrast, pavement-cell detection was more strongly species- and architecture-dependent, with reduced performance in monocot and highly divergent epidermal patterns. These findings indicate that broader application of *Stomatalia* to monocot pavement-cell analysis or divergent epidermal architectures will require additional annotation and model retraining.

**Fig. 6:**
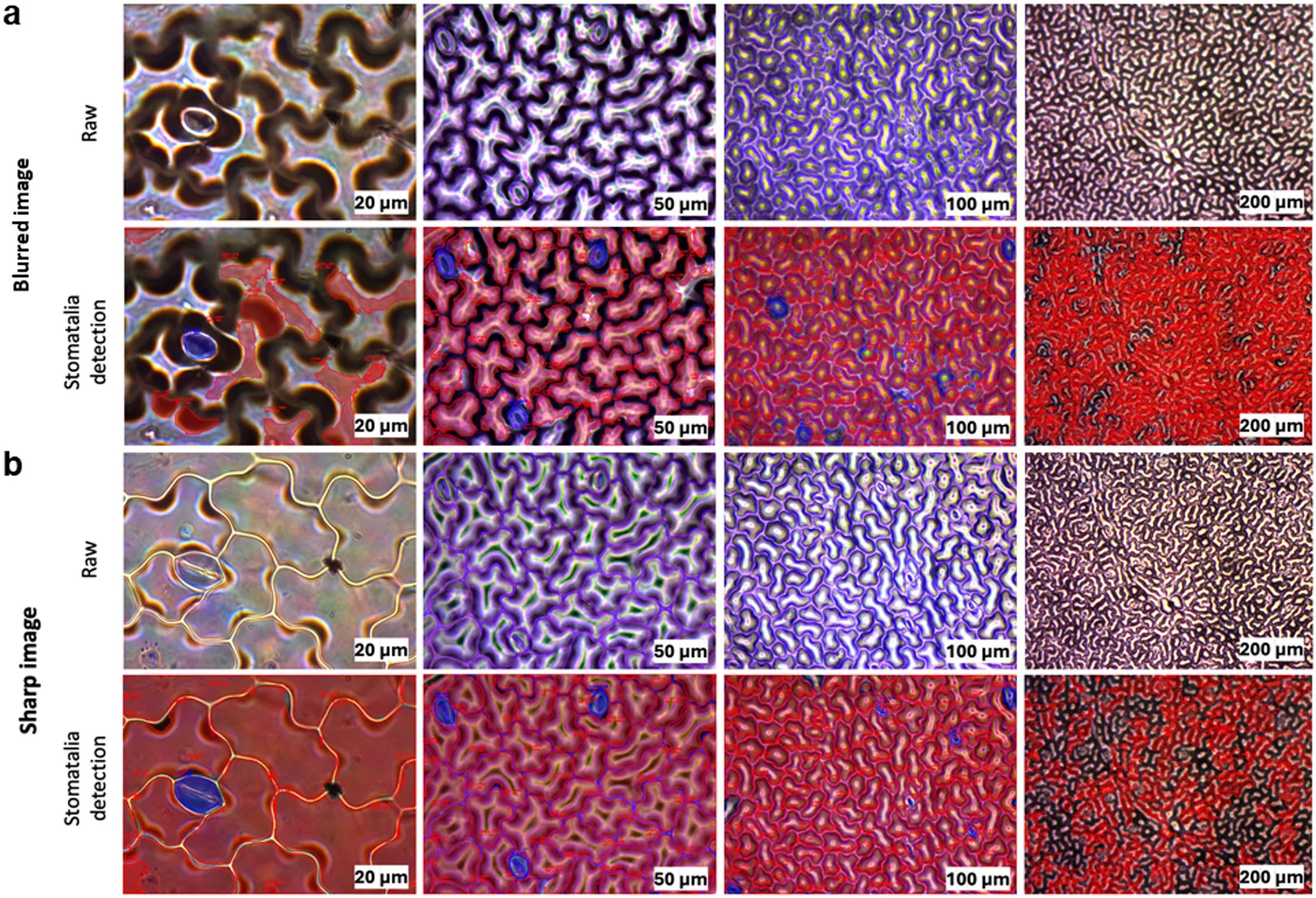
Representative raw images and corresponding *Stomatalia* detection outputs under varying image quality and magnification conditions. **a** Blurred images acquired at 20, 50, 100, and 200 μm scales. **b** Sharp images acquired at the same magnifications. Red overlays indicate detected pavement cells, whereas blue overlays indicate detected stomata

In addition to scale and species effects, the impact of image quality on detection performance was also assessed across magnifications (Fig. 6).

In sharp images, both stomata and pavement cells were accurately delineated, demonstrating the model’s ability to capture complex interlocking cell contours. Minor localised blurring led to occasional missed detections of pavement cells, while stomatal detection remained relatively stable. In contrast, extensive blurring significantly reduced detection of pavement cell boundaries, although stomatal detection was partially preserved. These results indicate that image sharpness is especially important for reliable pavement-cell quantification, while stomatal detection is more tolerant of moderate reductions in image quality.

**Fig. 7:**
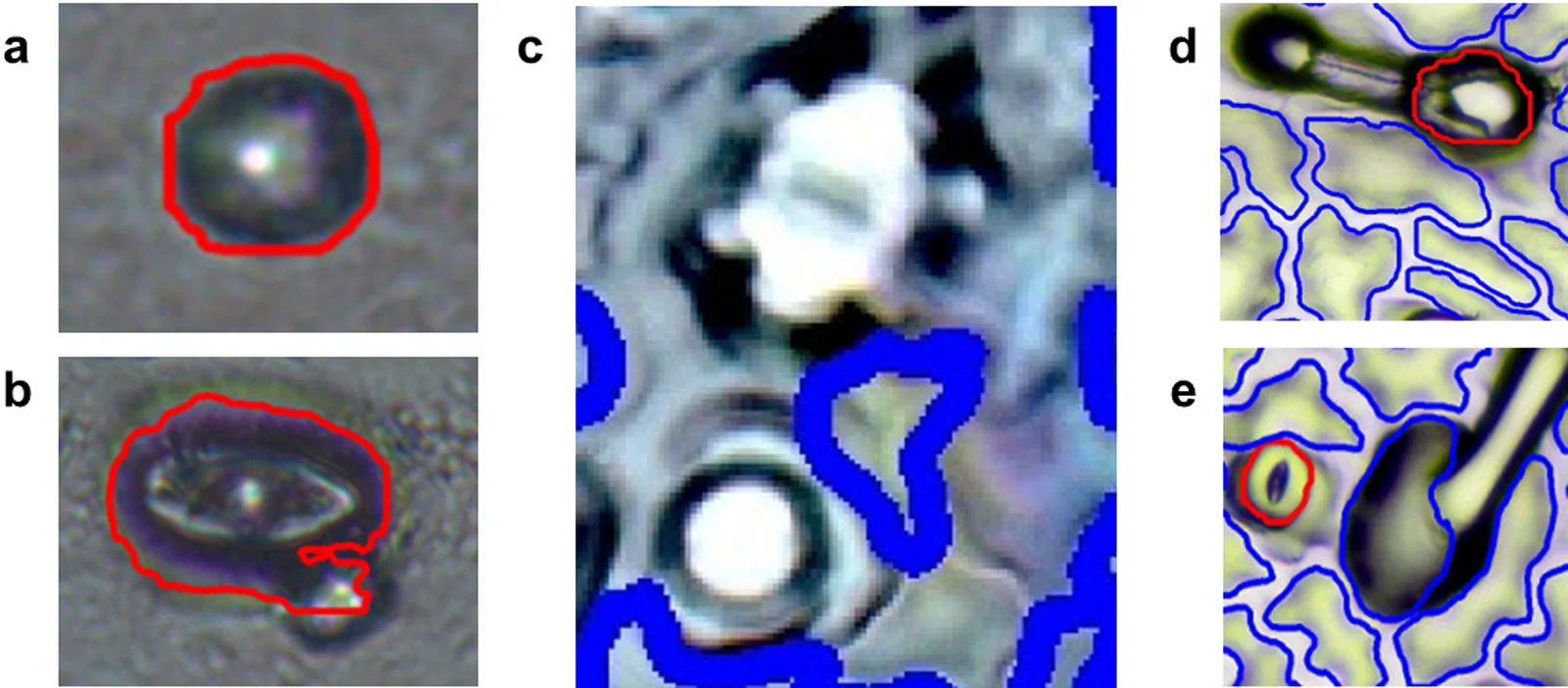
Classification of representative errors obtained using *Stomatalia*. **a** False-positive stomatal detection in a spring onion sample acquired at 20 μm. **b** Over-segmentation of a detected stomatal structure in a spring onion sample acquired at 20 μm. **c** Missed stomatal detection in a blurred tomato image acquired at 100 μm. **d** False-positive stomatal detection caused by a trichome in a tomato sample acquired at 50 μm. **e** False-positive pavement-cell detection associated with a trichome-like structure in a tomato sample acquired at 50 μm

A more detailed classification of representative detection errors is shown in Fig. 7.

In spring onion and other monocot or cross-domain images, occasional false-positive stomatal detections and over-segmentation of stomatal structures were observed (Fig. 7a, b), reflecting the effect of applying the model to an epidermal architecture outside its main training domain. In tomato images acquired at lower effective resolution or affected by blur, missed stomatal detections were more frequent, particularly at 100 μm (Fig. 7c). Additional false positives were associated with epidermal artefacts, such as trichomes, which were occasionally misclassified as stomata or pavement cells when their shape or contrast resembled the target structures (Fig. 7d, e). These observations indicate that *Stomatalia* relies strongly on high-frequency edge and boundary information for accurate instance segmentation, particularly for pavement cells. Therefore, maintaining image sharpness is critical for reliable trait extraction. While stomatal detection appears comparatively robust to moderate image degradation, pavement-cell quantification is more sensitive to loss of boundary contrast.

Overall, the quantitative and qualitative analyses show that *Stomatalia* provides reliable stomatal and pavement-cell outputs when applied to good-quality bright-field images within its validated dicot imaging domain. The external cross-species tests further indicate that stomatal detection can generalise to some additional dicot species, such as chickpea and balsam poplar, but performance is more variable in monocots and in species with divergent epidermal architectures. Broader application to monocot pavement-cell analysis or highly divergent epidermal types will therefore require additional annotation and model retraining.

### Web application deployment

**Fig. 8:**
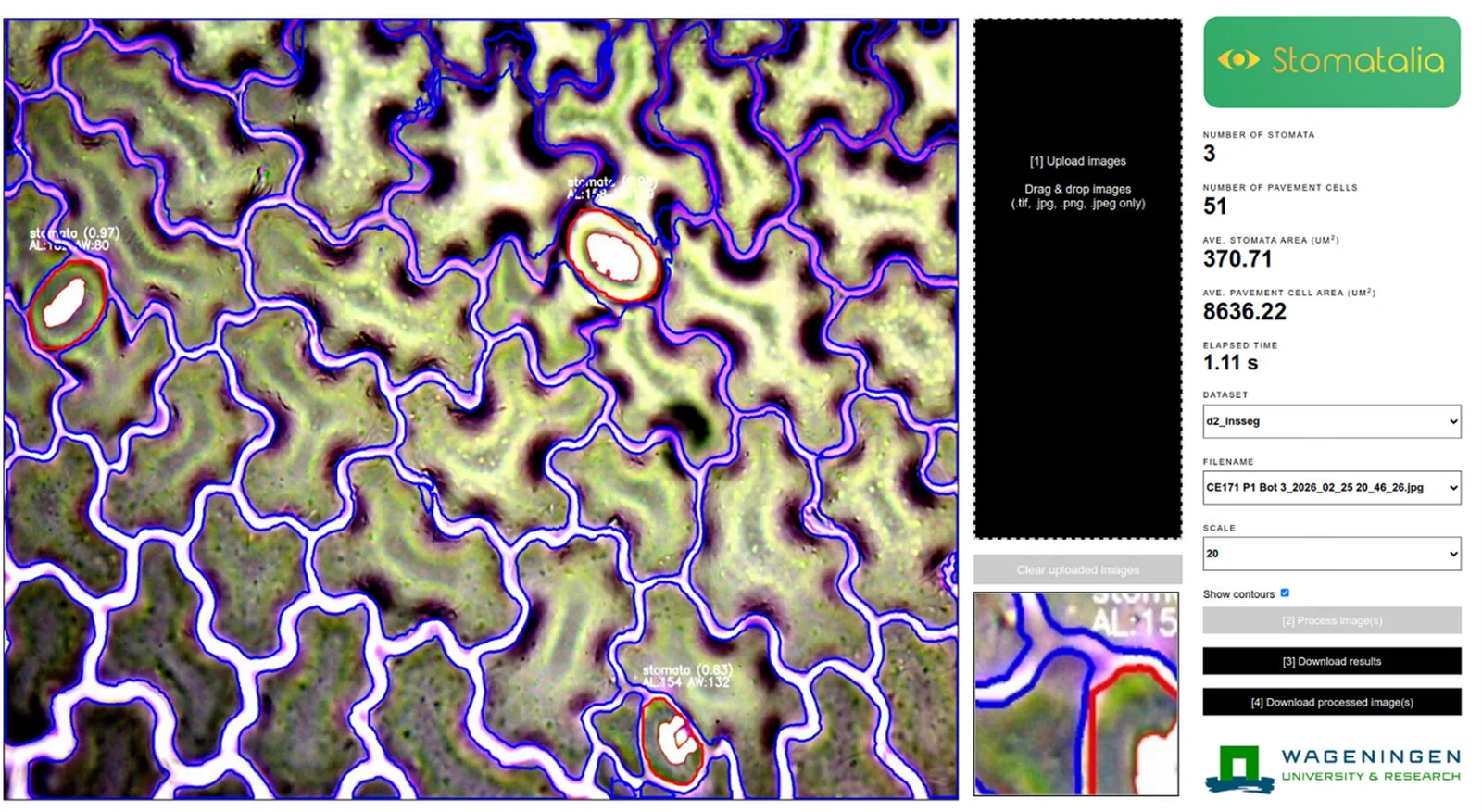
Screenshot of the *Stomatalia* web application interface. The platform enables image upload, scale selection, automated instance segmentation, visualisation of detected stomata and pavement cells, and export of quantitative trait data

The optimised instance segmentation model was deployed as a Flask-based web application (v2.3.1), named *Stomatalia* (Fig. 8). The platform enables browser-based analysis of leaf epidermal images without requiring local installation of computational dependencies.

Users upload images in .tif, .tiff, .png, .jpeg, or .jpg formats and specify the image scale to apply the appropriate pixel-to-micrometre conversion factor. Upon execution by pressing the *Process image(s)* button, the application performs automated instance segmentation and trait extraction using the deployed deep learning model. Processed images with overlaid detections can be visualised directly in the interface, and the output data, including raw measurements, derived traits, and metadata, can be downloaded in .xlsx format for downstream analysis. In addition, the original raw images remain accessible after analysis, allowing researchers to manually validate automated detections and confirm stomatal counts, thereby ensuring transparency and facilitating reproducibility for publication purposes. At the time of writing, *Stomatalia’s* source code and algorithm are distributed under a Creative Commons Attribution-ShareAlike 4.0 International license, allowing reuse, adaptation, and redistribution with appropriate credit and under the same license terms. To facilitate testing and reuse, we also provide a Google Colab demo at https://tinyurl.com/bdzea4pr, which includes a sample image-upload and processing app to test the *Stomatalia* algorithm.

### Comparability to available tools

Several tools provide powerful solutions for stomata or pavement-cell analysis. But their aims, input requirements, and biological scope differ from those of *Stomatalia*. StomataCounter, for example, was developed for stomatal detection in scanning electron microscopy and differential interference contrast images, rather than bright-field leaf prints [5]. PaCeQuant, PlantSeg, Cellpose, and MorphoGraphX support pavement-cell segmentation, but they are generally restricted to specific image-acquisition settings or cell morphologies and do not provide integrated stomatal trait extraction. For example, PaCeQuant and PlantSeg are primarily suited to well-defined confocal or bright-field datasets, with PlantSeg’s detection and segmentation accuracy limited to images taken under a light microscope (no UV) [13, 21]. Cellpose performance depends strongly on cell shape and is less reliable for highly interdigitated (puzzle-shaped) pavement cells [18]. Meanwhile, MorphoGraphX is a platform for surface segmentation of confocal 3D image stacks and is not compatible with brightfield imaging [1]. These tools remain valuable, but their specialisation highlights the need for complementary workflows that jointly quantify stomata and pavement cells from high-throughput 2D bright-field datasets.

In this context, LeafNet is the available platform that most closely overlaps with *Stomatalia*, as it combines stomatal localisation and pavement-cell segmentation in bright-field images and 2D images extracted from 3D stacks [10]. Moreover, LeafNet’s research study reports extensive benchmark analyses against StomataCounter for stomata detection and against PlantSeg, PaCeQuant, and MorphoGraphX for pavement-cell segmentation [10]. Thus, we considered LeafNet the most suitable comparator for evaluating *Stomatalia* against an existing integrated epidermal-analysis workflow.

This benchmark analysis was performed through the web interfaces of both *Stomatalia* and LeafNet, using manual ground-truth counts as reference and recording processing time *per* image. The comparison included potato leaf-print images, representing a dicot epidermal architecture within *Stomatalia’s* validated domain; spring onion images, representing a monocot epidermis outside *Stomatalia’s* main training domain; and *Arabidopsis thaliana* Col-0 images, included because LeafNet was originally trained with bright-field images of Arabidopsis leaf surfaces. All raw and output detection images, along with the result files, are available via Figshare (https://doi.org/10.6084/m9.figshare.32532672), and the benchmark summary table is provided in Supplementary file 1.

As summarised in Table 7, *Stomatalia* closely matched manual counts for both stomata and pavement cells in potato images, with mean values of 11.7 stomata and 68.2 pavement cells *per* image compared with manual ground-truth values of 12.2 and 68.2, respectively. In contrast, the default LeafNet workflow returned no stomatal detections for the potato images and overestimated the number of pavement cells. A similar pattern was observed in *A. thaliana* Col-0: *Stomatalia* underestimated stomatal number but remained close to the manual pavement cell counts, whereas the default LeafNet workflow again returned zero stomata and strongly overestimated pavement cell number. In spring onion, *Stomatalia* detected stomata with counts close to the manual ground truth, but, as expected, did not reliably segment the pavement cells, consistent with the current model being primarily validated for dicot epidermal architectures. LeafNet also returned zero stomatal detections for spring onion under the default workflow and produced highly variable pavement cell counts, particularly when the image-resolution parameter was lowered to 0.54 px/µm.

Processing time further distinguished *Stomatalia* and LeafNet web-based workflows. *Stomatalia* processed images in approximately 0.4–2.1 s *per* image, depending on the dataset, whereas LeafNet required several minutes *per* image under standard settings and more than 7200 s *per* image when spring onion images were analysed at 0.54 px/µm. Together, these results indicate that *Stomatalia* provides a rapid and accurate workflow for coordinated stomatal and pavement-cell analysis within its validated dicot bright-field leaf-print domain. At the same time, the benchmark defines clear limitations: performance decreases in lower-quality or lower-resolution images, and reliable pavement-cell segmentation in monocots or species with strongly divergent epidermal architectures will require additional annotation and model retraining. *Stomatalia* should therefore be viewed as complementary to existing tools rather than as a universal replacement, with particular value for studies requiring integrated interpretation of stomatal traits in relation to epidermal expansion.

**Table 7:** Benchmark comparison between manual ground truth, *Stomatalia*, and LeafNet across independent test images

Dataset		*n*	LeafNet image	*N*_S_					*N*_PC_					Time/image	
			Resolution	Ground truth	*Stom*	MAE	LeafN	MAE	Ground truth	*Stom*	MAE	LeafN	MAE	Stom	LeafN
Potato		9	2.17 px/µm	12.2	11.7	0.6	0	12.2	68.2	68.2	0	235.1	166.8	2.09 s	314.5 s
Spring onion		6	2.17 px/µm	6.2	5.7	0.5	0	6.2	16.8	0	16.8	135.6	118.8	0.40 s	408.2 s
Spring onion		3	0.54 px/µm	6.3	6	0.3	0	6.3	16	0.3	15.7	1973.5	1957.5	0.42 s	7254.7 s
*A. thaliana* Col-0		9	1.74 px/µm	21	15.1	5.9	0	21	79.6	74.7	5.3	1300.3	1220.7	2.08 s	348.9 s

Values are means per image. LeafNet was run through the web interface using the default workflow unless otherwise stated

Stom: *Stomatalia*; LeafN: LeafNet; N_S_: number of stomata; N_PC_: number of pavement cells; MAE: mean absolute error compared with manual ground truth

### Experimental validations

**Fig. 9:**
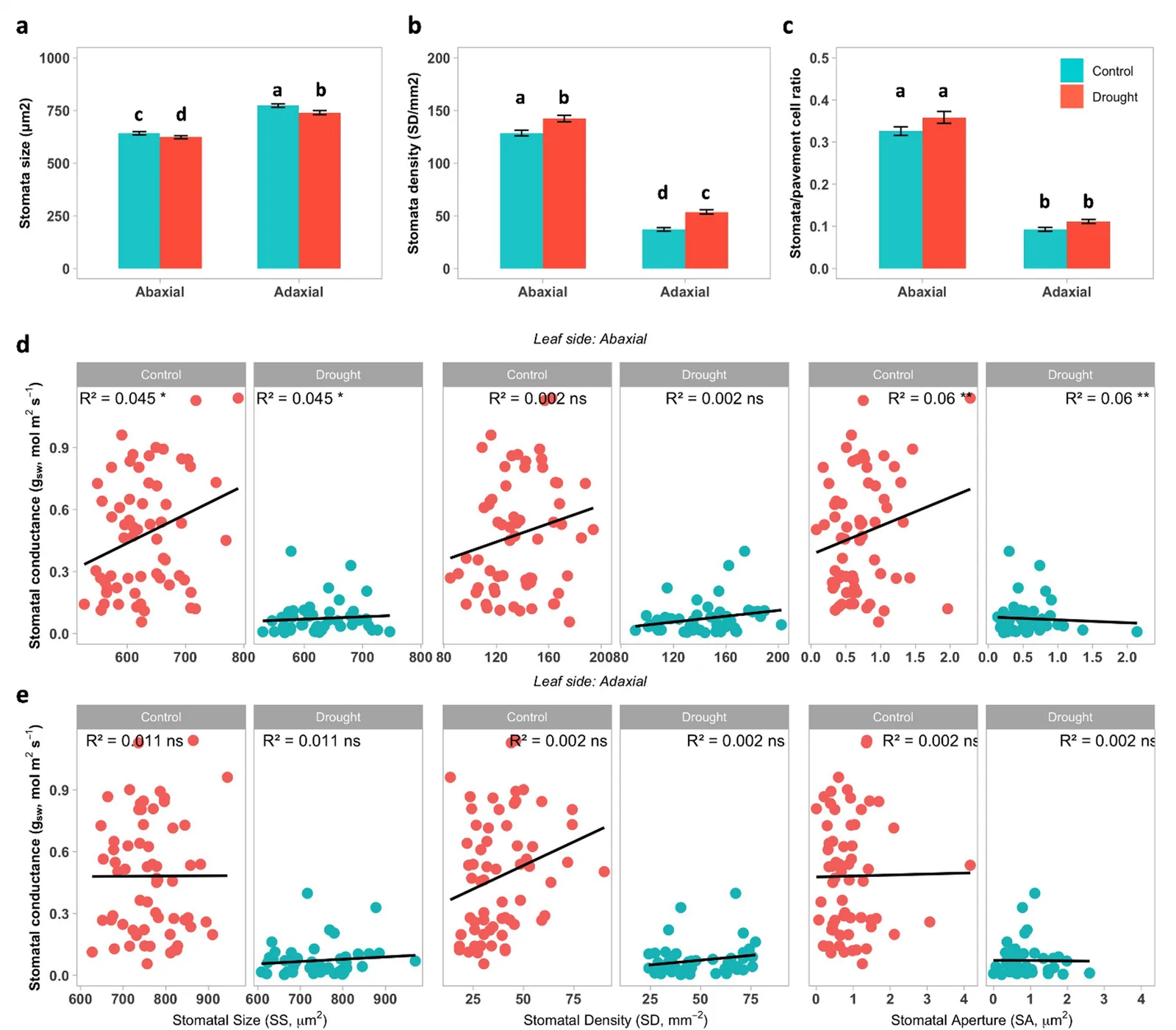
Changes in stomatal and pavement cell traits extracted by *Stomatalia *from potato cultivars subjected to drought and well-irrigated conditions. **a** Stomatal size (SS, µm^2^). **b** Stomatal density (SD, stomata mm^2^). **c** Stomata-to-pavement cell ratio. Correlations between stomatal conductance (gsw, mol m^2^ s^− 1^) and stomatal size, density, and aperture (SA, µm^2^) are shown for **d** abaxial and **e** adaxial leaf surfaces. Error bars represent standard errors of the mean. Different letters indicate significant differences (*P* ≤ 0.05; ANOVA with Tukey’s post hoc test). Asterisks denote significant linear regressions (*P* < 0.001), and ns indicates non-significance

*Stomatalia’s* applicability under biologically relevant conditions was evaluated through a proof-of-concept drought trial in cultivated potato. After seventeen days of treatment, 192 leaf-print images from abaxial and adaxial surfaces were processed using *Stomatalia* to quantify drought-induced changes in stomatal and pavement cell traits (Fig. 9a–c).

Drought significantly reduced stomatal size and increased stomatal density on both leaf surfaces. At first glance, these results suggest a structural shift towards smaller, denser stomata, a pattern frequently interpreted as an adaptive response to water limitation. During prolonged drought, newly developed leaves often exhibit reduced stomatal size and altered stomatal density as adaptations to reduce transpirational water loss [14]. While reducing stomatal size helps conserve water, it also restricts its uptake, limiting photosynthetic capacity and overall growth. Stomatal traits are therefore frequently assessed in drought studies; however, their interpretation is often complicated by artefacts arising from reduced leaf expansion [11].

*Stomatalia* addresses this challenge by simultaneously quantifying stomatal and pavement cell traits, enabling the distinction between true developmental changes and biased findings. Interestingly, analysis of the stomata-to-pavement cell ratio revealed no significant differences between treatments for either leaf surface (Fig. 9c). This result indicates that drought-induced modifications detected for stomata were paralleled by changes in pavement cells. Therefore, in response to water deficit, pavement cell size was also reduced, which may explain the apparent increase in stomatal density. Reduced epidermal cell expansion under drought is consistent with lower turgor pressure and assimilate availability [4, 19]. Thus, the observed stomatal patterns likely reflect growth limitation rather than specific reprogramming of stomatal development. This distinction is critical, as the risk of misinterpreting stomatal density and size alone has been reported in the drought literature [3, 22].

Assessing stomatal morphology without accounting for concomitant changes in epidermal expansion can therefore obscure the identification of true developmental adjustments or at least limit their biological interpretation. To address these limitations, *Stomatalia* provides a comprehensive, physiologically meaningful platform for studying coordinated changes in stomatal and pavement cell traits across dicot species and environmental stresses, such as drought.

When combined with gas-exchange measurements, anatomical traits provided by *Stomatalia* can help disentangle structural capacity from functional control of transpiration, offering a more integrated interpretation of plant responses. However, no strong or biologically meaningful associations were detected between stomatal size, density, or aperture and stomatal conductance (Fig. 9d, e). This lack of association was consistent across leaf surfaces and treatments. While developmental plasticity in stomatal patterning can influence the structural capacity for gas exchange [2], our results indicate that, in these cultivated potato genotypes, short-term functional regulation of stomatal conductance was not strongly influenced by morphological variation.

These findings do not undermine the relevance of stomatal development in stress adaptation. Rather, they highlight that within cultivated germplasm with limited genetic diversity, structural adjustments may play a comparatively minor role relative to dynamic stomatal regulation. Crucially, such conclusions can only be drawn when anatomical and physiological traits are quantified and interpreted in an integrated framework, precisely the analytical capacity enabled by *Stomatalia*.

Collectively, these results illustrate how integrating stomatal and pavement cell traits refines the interpretation of stress-induced anatomical changes. Beyond accelerating phenotyping, *Stomatalia* provides a framework for distinguishing growth-related artefacts from genuine developmental adaptations.

### Final reflections

We developed *Stomatalia*, a robust, user-friendly deep-learning platform for automated, high-throughput analysis of leaf epidermal images across varying magnifications and resolutions. The platform enables accurate, rapid, and reproducible phenotyping of stomatal and pavement cell traits within a unified analytical workflow.

Algorithm validation identified key technical considerations that should guide its use. While lower magnifications increase the observable leaf area *per* image, detection accuracy declines as the field of view increases, particularly beyond 50 μm scale. We therefore recommend image acquisition at 20–50 μm scales to balance coverage and precision. Image quality was also critical, especially for pavement-cell segmentation: blurred regions substantially reduce boundary detection, whereas stomatal detection remains comparatively robust. Users primarily interested in stomatal traits may therefore tolerate lower image quality, but pavement cell data derived from blurred or low-contrast images should be interpreted with caution.

The cross-species feasibility test further defined the model’s current domain. *Stomatalia* was trained and validated primarily on dicotyledonous species with sinuous pavement-cell morphology, such as tomato and potato. Consistent with this, the most reliable combined stomatal and pavement-cell outputs were obtained within this dicot image domain. Stomatal detection nevertheless generalised to several additional species, including spring onion, chickpea, balsam poplar, and wheat, although performance was more variable in monocots and in species with divergent epidermal architectures. In contrast, pavement-cell segmentation was more species- and architecture-dependent, with reduced performance in monocots and some external datasets. These results indicate that *Stomatalia* is not a universal epidermal segmentation tool in its current form, but with additional retraining and targeted annotation, its framework can be extended to additional taxa.

Benchmarking against LeafNet provided additional practical context. LeafNet is the existing platform that most closely overlaps with *Stomatalia* because it combines stomatal localisation and pavement-cell segmentation from bright-field epidermal images. In the tested web-based workflows, *Stomatalia* showed closer agreement with manual counts for the evaluated images of potato and *Arabidopsis thaliana* Col-0 and processed them substantially faster. However, this comparison should be interpreted in the context of the tested image types, parameter settings, and web interface conditions. Rather than positioning *Stomatalia* as a replacement for existing tools, these results highlight its value as a complementary workflow for high-throughput analysis of leaf prints, particularly when coordinated extraction of stomatal and pavement-cell traits is required.

Importantly, the proof-of-concept trial demonstrated that *Stomatalia* facilitates integrated analysis of anatomical and physiological traits. Assessing stomatal morphology in isolation can lead to misleading conclusions, particularly under stress conditions that also constrain leaf expansion. We therefore strongly recommend integrating pavement-cell metrics when interpreting stomatal density and size, especially in studies addressing stress physiology, growth dynamics, or breeding for water-use efficiency. By integrating pavement-cell measurements with stomatal data, *Stomatalia* enables a more accurate interpretation of whether anatomical responses reflect stress-induced growth limitation, altered epidermal expansion, or genuine adaptations in stomatal development.

Overall, *Stomatalia* provides a versatile and extensible framework for quantitative epidermal phenotyping from 2D leaf-print images. Its strongest current application is rapid, coordinated analysis of stomatal and pavement-cell traits in high-quality dicot leaf impressions, while future expansion to monocots and more divergent epidermal architectures will require additional annotated datasets. Within these defined boundaries, *Stomatalia* advances research on epidermal plasticity, environmental adaptation, and crop improvement under changing climatic conditions.

## Supplementary Information

Below is the link to the electronic supplementary material.

Supplementary Material 1.

## Data Availability

Stomatalia © 2026 by Wageningen University & Research is licensed under Creative Commons Attribution-ShareAlike 4.0 International. To view a copy of this license, visit https://creativecommons.org/licenses/by-sa/4.0/. The source code and algorithm of Stomatalia are distributed under the aforementioned license, which requires reusers to credit the creator. It allows reusers to distribute, remix, adapt, and build upon the material in any medium or format, even for commercial purposes. If others remix, adapt, or build upon the material, they must license the modified material under identical terms.To facilitate testing and reuse, a Google Colab demo is available at https://tinyurl.com/bdzea4pr, which includes a sample image-upload and processing app to test the Stomatalia algorithm. The Colab notebook does not feature the full version of the Stomatalia app, but it allows users to analyse images.The test datasets and model file used in this work are available through the following link: https://doi.org/10.6084/m9.figshare.32532672. The test_sets.zip archive contains the test image datasets (cross-species and benchmark analysis), including raw/input images, detection output images, manual ground-truth counts and exported datasets. A README file is provided to describe the content and intended use of the deposited files. Filenames have been anonymised to ensure confidentiality of genotype information. Additional datasets not included in the public archive are available from the corresponding authors upon reasonable request.
